# Morphometric, Hemodynamic, and Multi-Omics Analyses in Heart Failure Rats with Preserved Ejection Fraction

**DOI:** 10.3390/ijms21093362

**Published:** 2020-05-09

**Authors:** Wenxi Zhang, Huan Zhang, Weijuan Yao, Li Li, Pei Niu, Yunlong Huo, Wenchang Tan

**Affiliations:** 1 Department of Mechanics and Engineering Science, College of Engineering, Peking University, Beijing 100871, China; wenxiz@pku.edu.cn (W.Z.); lili2016@pku.edu.cn (L.L.); niupei@pku.edu.cn (P.N.); 2 Hemorheology Center, Department of Physiology and Pathophysiology, School of Basic Medical Sciences, Peking University Health Science Center, Beijing 100191, China; zhanghuansoul@163.com (H.Z.); weijuanyao@bjmu.edu.cn (W.Y.); 3 Institute of Mechanobiology & Medical Engineering, School of Life Sciences & Biotechnology, Shanghai Jiao Tong University, Shanghai 200240, China; 4 PKU-HKUST Shenzhen-Hongkong Institution, Shenzhen 518057, China; 5 Shenzhen Graduate School, Peking University, Shenzhen 518055, China

**Keywords:** heart failure with preserved ejection fraction, speckle tracking echocardiography, left ventricular hypertension, myocardial remodeling, multi-omics analysis

## Abstract

(1) Background: There are no successive treatments for heart failure with preserved ejection fraction (HFpEF) because of complex interactions between environmental, histological, and genetic risk factors. The objective of the study is to investigate changes in cardiomyocytes and molecular networks associated with HFpEF. (2) Methods: Dahl salt-sensitive (DSS) rats developed HFpEF when fed with a high-salt (HS) diet for 7 weeks, which was confirmed by in vivo and ex vivo measurements. Shotgun proteomics, microarray, Western blot, and quantitative RT-PCR analyses were further carried out to investigate cellular and molecular mechanisms. (3) Results: Rats with HFpEF showed diastolic dysfunction, impaired systolic function, and prolonged repolarization of myocytes, owing to an increase in cell size and apoptosis of myocytes. Heatmap of multi-omics further showed significant differences between rats with HFpEF and controls. Gene Set Enrichment Analysis (GSEA) of multi-omics revealed genetic risk factors involved in cardiac muscle contraction, proteasome, B cell receptor signaling, and p53 signaling pathway. Gene Ontology (GO) analysis of multi-omics showed the inflammatory response and mitochondrial fission as top biological processes that may deteriorate myocyte stiffening. GO analysis of protein-to-protein network indicated cytoskeleton protein, cell fraction, enzyme binding, and ATP binding as the top enriched molecular functions. Western blot validated upregulated Mff and Itga9 and downregulated Map1lc3a in the HS group, which likely contributed to accumulation of aberrant mitochondria to increase ROS and elevation of myocyte stiffness, and subsequent contractile dysfunction and myocardial apoptosis. (4) Conclusions: Multi-omics analysis revealed multiple pathways associated with HFpEF. This study shows insight into molecular mechanisms for the development of HFpEF and may provide potential targets for the treatment of HFpEF.

## 1. Introduction

The prevalence of heart failure (HF) with preserved ejection fraction (HFpEF), whose morbidity, mortality, and healthcare costs are similar to HF with reduced EF (HFrEF), is rising throughout the world [1,2]. The diastolic left ventricular (LV) dysfunction seen in HFpEF is mainly characterized by slow LV relaxation and elevated diastolic LV stiffness [3]. Despite impairment of regional systolic functions, global preservation of systolic function has been shown in several studies [4,5,6]. Abnormal ventricular–arterial coupling, pulmonary hypertension, renal insufficiency, vascular dysfunction, and skeletal muscle abnormalities also occur in patients with HFpEF [2,7]. These pathophysiological changes are associated with many determinants including extracellular matrix abnormalities, oxidative balance, myocyte stiffness, inflammation, altered myocardial energetics, and so on [1,2]. Although substantial molecular pathways lead to these changes in organ and tissue levels [8], there is still lack of successful treatments for HFpEF given the complexity of molecular networks. As a logical starting point, one aim of the study is to investigate the molecular networks related to cardiac abnormalities in HFpEF based on a multi-omics analysis.

Dahl salt-sensitive (DSS) rat model is a mutant strain of Sprague–Dawley rats characterized by hypersensitivity to sodium intake [9]. This model easily develops HFpEF when placed on a high-salt (HS) diet and is suitable for the study of molecular pathways and mechanisms in HFpEF, albeit it only represents some patients suffering from salt-sensitive hypertension [10,11]. In previous studies, increased oxidative stress and ROS production were observed in various organs including the kidney [12,13], heart [14,15], and blood vessels [16] in DSS. Excess ROS may contribute to impairment of LV diastolic function through Ca^2+^-handling proteins [17]. Considerable attention was paid to the attenuation of hypertension by antioxidant interventions in DSS. Cilnidipine could attenuate the overload-induced increases in ROS levels and contribute to the greater amelioration of LV diastolic dysfunction in the heart of DSS [18]. Hydrogen sulfide could improve aortic structural remodeling and reduce high salt-induced renal injury in DSS [19]. High-salt diet increased cerebrospinal fluid (CSF) and caused sympathetic hyperactivity and hypertension [20,21]. A chronic increase in CSF by high-salt diet in DSS increases hypothalamic tissue aldosterone and endogenous ouabain [22,23].

This study carried out morphological, hemodynamic, electrocardiogram, and multi-omics analyses on the heart tissues of DSS rats fed with a HS diet as compared with the control DSS rats, which were placed on a low-salt (LS) diet. We found that HS-fed DSS rats developed HFpEF, characterized by diastolic dysfunction, impaired systolic function, and prolonged repolarization of myocytes. The multi-omics analyses revealed multiple pathways that may be strongly associated with HFpEF, such as cardiac muscle contraction, inflammatory response, mitochondrial fission, and cytoskeleton protein. Western blot and real-time PCR were performed to validate the dysregulation of several genes, such as Mff, Map11c3a, Integrin α9, Plcb2, Diaph3, Btk, Tlk7, and so on. The significance and implications of our study could improve our understanding of the molecular mechanisms for the development of HFpEF and may provide potential targets for the treatment of HFpEF.

## 2. Results

### 2.1. Development of HFpEF in HS-Fed Rats

**Figure 1:**
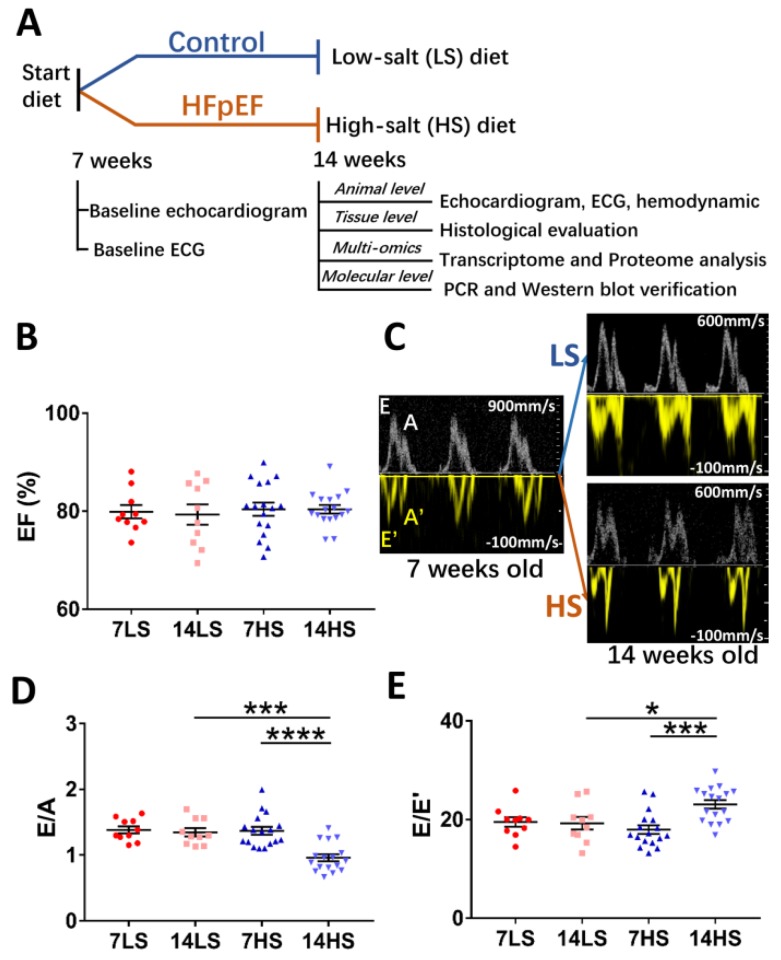
Experimental protocol and evaluation of heart function of Dahl salt-sensitive (DSS) rats. (**A**) A schematic representative of experimental protocol, where 7-week-old Dahl salt-sensitive (DSS) rats were fed a high-salt (*n* = 17) or low-salt diet for 7 weeks (*n* = 10). (**B**) Ejection fraction (EF (%) mean ± SE) in the two groups at the age of 7 and 14 weeks. (**C**) A schematic representative of Doppler-mode tracings in rat hearts, and (**D**) E/A and (**E**) E/E’ in the two groups at the ages of 7 and 14 weeks. * *p* < 0.05, *** *p* < 0.001, **** *p* < 0.0001.

**Figure 2:**
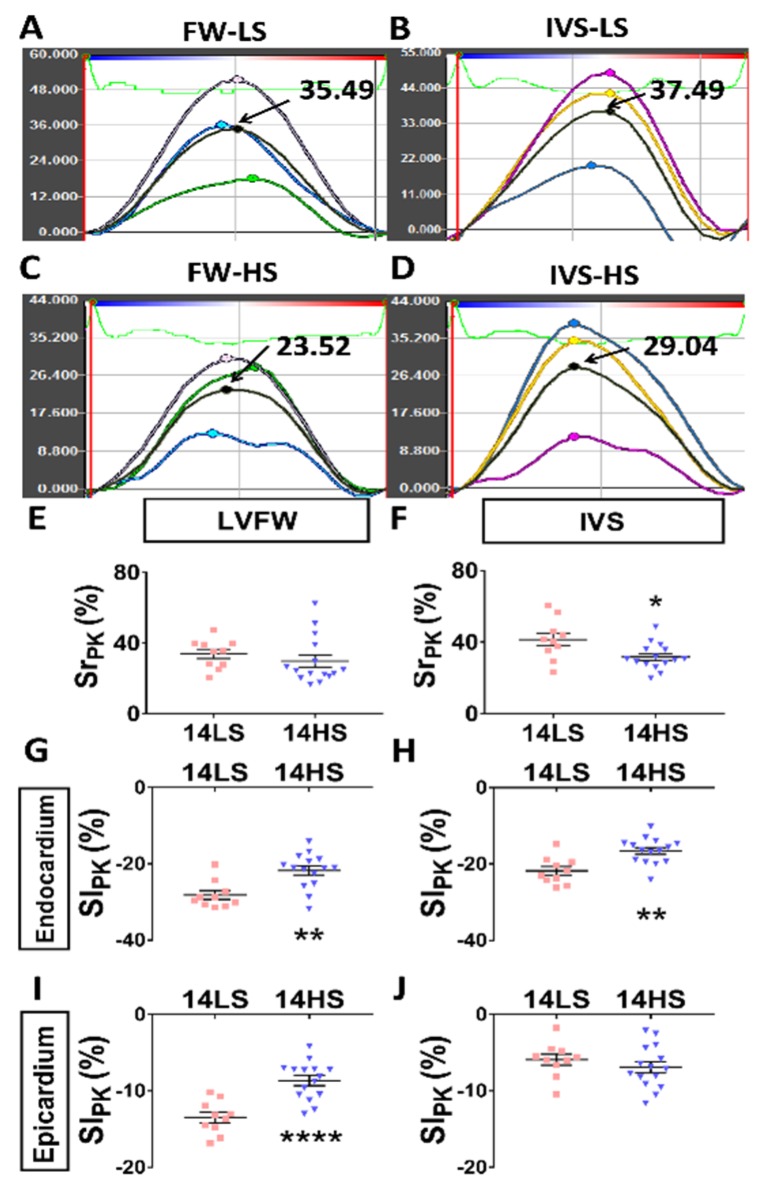
Severely impaired systolic contractile function in free wall (FW) and interventricular septum (IVS) of high-salt (HS)-fed DSS rat hearts. (**A**–**D**) Representative images of radial strain curves in FW and IVS in low-salt (LS)-fed and HS-fed rat hearts at the age of 14 weeks. The color lines represent the radial strain curves corresponding to the six myocardial segments, and the black line refers to the averaged strain curve over free wall or interventricular septum, where the arrow points to the peak value. (**E** and **F**) Peak radial strains in FW and IVS in LS-fed and HS-fed rat hearts. (**G**–**J**) Peak longitudinal strains on the endocardium and epicardium of FW and IVS in LS-fed and HS-fed rat hearts (*n* = 10 in LS group and *n* = 15 in HS group). * *p* < 0.05, ** *p* < 0.01, **** *p* < 0.0001.

DSS rats 7 weeks old were fed with HS or LS diet for 7 weeks. Their heart functions were evaluated by echocardiogram, electrocardiogram (ECG), and hemodynamics (Figure 1A). The values of EF (%), E/A, and E/E’ in HS and LS groups were obtained at 7 weeks (baseline) and 14 weeks. Data showed that EF was preserved in both groups (Figure 1B). The E/A and E/E’ ratios were equivalent in the two groups at baseline (Figure 1C and 1D). No change was found in E/A and E/E’ ratios at 14 weeks in the LS-fed group. However, in the HS-fed group, E/A ratio was significantly decreased (0.96 ± 0.05 in HS vs. 1.35 ± 0.06 in LS, *p* < 0.001), while E/E’ ratio (23.05 ± 0.85 in HS vs. 19.25 ± 1.26 in LS, *p* < 0.05) was significantly increased at 14 weeks (Figure 1D). Diastolic dysfunction was defined as either E/A ratio < 1.29 or E/E’ > 20.51 according to the 95% distribution values of E/A and E/E’ of 10 LS-fed rats at 14 weeks of age. HS-fed rats were verified to have developed diastolic dysfunction. These rats also showed signs of HF such as weakness and decreased activity and so were classified to have HFpEF. Moreover, Figure 2A–D shows representative images of radial strain curves in free wall (FW) and interventricular septum (IVS) in LS-fed and HS-fed rat hearts. Figure 2E,F shows peak radial strains in FW and IVS, while Figure 2G–J shows peak longitudinal strains on endocardium (endo) and epicardium (epi) of FW and IVS. Data showed that HS-feeding resulted in a decrease of peak radial (FW: 29.79 ± 3.49 in HS vs. 33.94 ± 2.61 in LS, *p* = 0.39; IVS: 31.77 ± 1.89 in HS vs. 41.52 ± 3.57 in LS, *p* < 0.05) and longitudinal strains in the LV except for the epicardium of IVS (FW endo: −21.75 ± 1.18 in HS vs. −28.19 ± 1.12 in LS, *p* < 0.01; IVS endo: −16.58 ± 0.87 in HS vs. −21.77 ± 1.11 in LS, *p* < 0.01; FW epi: −8.69 ± 0.65 in HS vs. −13.49 ± 0.69 in LS, *p* < 0.0001; IVS epi: −6.93 ± 0.72 in HS vs. −5.92 ± 0.72 in LS, *p* = 0.35), which indicates severely impaired systolic contractile function. Table 1 lists morphometric differences between the two groups at the age of 14 weeks. HS feeding increased ratios of heart, lung, and kidney weights to BW and the LV end systolic and end diastolic diameters, which reveals myocardial hypertrophy, pulmonary congestion, and kidney impairment.

### 2.2. ECG Analyses in Control and HFpEF Rats 

**Figure 3:**
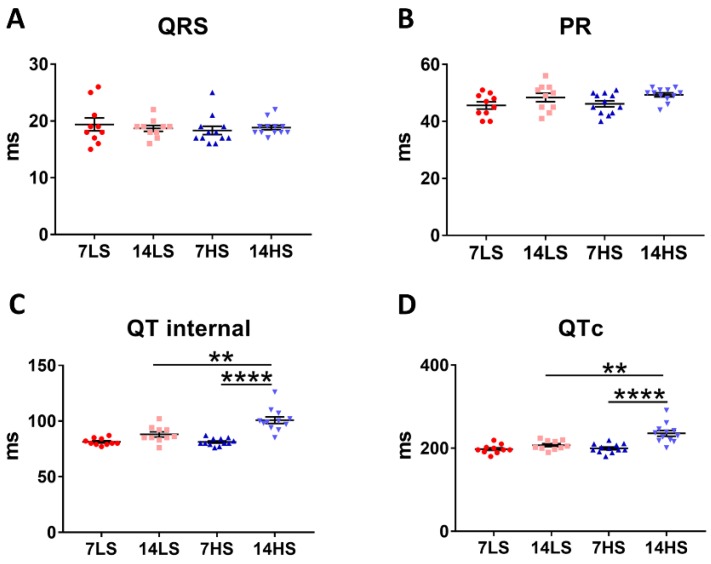
Electrocardiogram analysis showed prolonged myocyte repolarization in HS-fed DSS rat hearts. (**A**–**D**) QRS width, PR interval, QT interval, and QTc interval in the two groups at the ages of 7 and 14 weeks (*n* = 10 in LS group and *n* = 12 in HS group). ** *p* < 0.01, **** *p* < 0.0001.

Similar values of QRS width and PR interval between the two groups demonstrated no conduction delays in HS-fed rat hearts (Figure 3A,B). A significant increase of QT interval (100.8 ± 2.96, in HS vs. 88.0 ± 2.27 in LS, *p* < 0.01) and QTc interval (235.7 ± 6.81 in HS vs. 207.3 ± 3.60 in LS, *p* < 0.01) (i.e., the time from the onset of ventricular depolarization to the completion of repolarization) depicts the prolonged repolarization in HS-fed rat hearts (Figure 3C,D). 

### 2.3. Hemodynamic Analysis

Hemodynamic analysis showed the increased LV end diastolic pressure (LVEDP) (11.14 ± 2.03 mmHg in HS vs. 3.72 ± 0.38 mmHg in LS), decreased dP/dt max (7010 ± 941 mmHg/s in HS vs. 9895± 833 mmHg/s in LS), decreased absolute value of dP/dt min (5051 ± 489 mmHg/s in HS vs. 6890 ± 528 mmHg/s in LS), and elongated Tau value (15.21 ± 2.05 ms in HS vs. 9.40 ± 0.71 ms in LS) in HS-fed rat hearts as listed in Table 2. Aortic blood pressure was also elevated significantly including arterial systolic pressure (ASP) (184.6 ± 6.8 mmHg in HS vs. 160.5 ± 7.9 mmHg in LS), arterial diastolic pressure (ADP) (128.2 ± 4.4 mmHg in HS vs. 106.5 ± 7.7 mmHg in LS), and mean arterial pressure (MAP) (147.0 ± 4.9 mmHg in HS vs. 124.5 ± 6.9 mmHg in LS) as listed in Table 2. Dahl salt-sensitive rats fed the HS diet developed hypertension at 14 weeks of age. The mRNA levels of two hypertrophic genes, BNP and TIMP1, were found markedly elevated in HS-fed rats (Supplementary Table S2). All these pathophysiological changes as shown in Figure 1, Figure 2 and Figure 3 and Table 1 and Table 2 verified the occurrence of HFpEF in HS-fed DSS rat hearts.

### 2.4. Histological Evaluation

**Figure 4:**
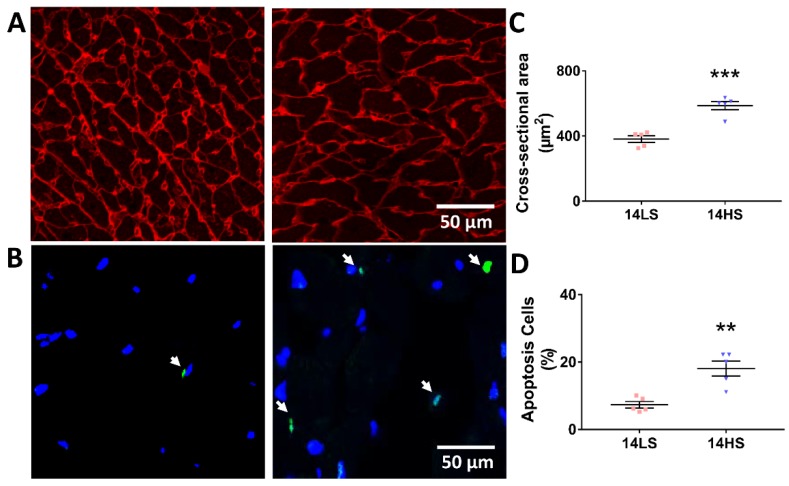
Histological evaluation showed increase in size and apoptosis of myocytes of HS-fed DSS rats. (**A**,**B**) Representative immunofluorescence images of myocytes stained by WGA and TUNEL (arrow refers to apoptotic cells) and (**C**,**D**) cross-sectional area of a myocyte and apoptosis rate (TUNEL-positive nuclei as percentage of total nuclei) in the two groups at the age of 14 weeks (*n* = 5 for each group). ** *p* < 0.01, *** *p* < 0.001.

Figure 4A,B shows representative images of myocytes stained by Wheat Germ Agglutinin (WGA) and terminal deoxynucleotidyl transferase dUTP nick end labeling (TUNEL) in HS and LS groups at the age of 14 weeks. The corresponding statistical results are presented in Figure 4C,D. The statistical analysis revealed that HS feeding led to a larger size of myocytes (587 ± 25.64 μm^2^ in HS vs. 381.4 ± 20.2 μm^2^ in LS, *p* < 0.001) as well as a significant increase of myocyte apoptosis rate (0.18 ± 0.022 in HS vs. 0.073 ± 0.0095 in LS, *p* < 0.01).

### 2.5. Proteomic Study and Western Blot

**Figure 5:**
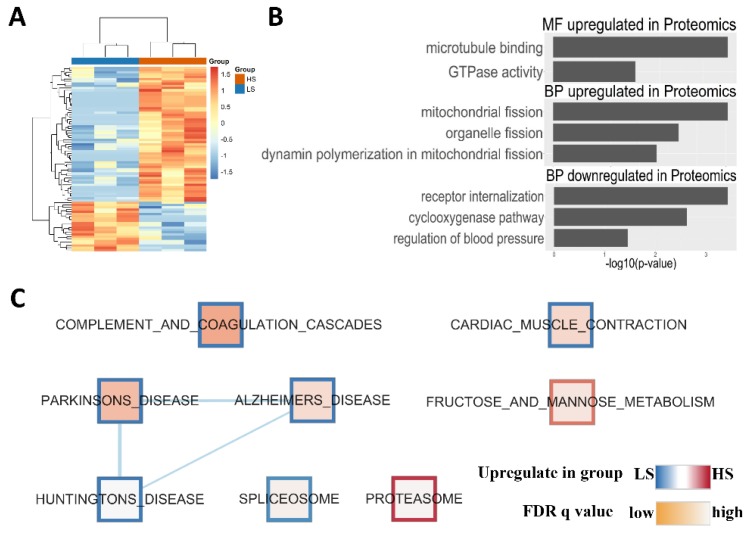
Gene Ontology (GO) and Gene Set Enrichment Analysis (GSEA) on dysregulated proteins identified by proteomics. (**A**) Heatmap of the proteome, where hierarchical clustering of samples (columns) and proteins (rows) is based on Pearson’s correlation coefficient to measure the distance and the mean to cluster the samples. (**B**) Gene Ontology (GO) analysis of 21 significantly downregulated and 59 significantly upregulated proteins. (**C**) Gene Set Enrichment Analysis (GSEA) of all detected proteins, where nodes represent significantly enriched (*p* < 0.01, *q* < 0.05) gene sets of canonical pathways from the molecular signature database (MSigDB). The colors of node borders refer to upregulated (red) and downregulated (blue) in HS group, while the node color represents the FDR *q*-values

**Figure 6:**
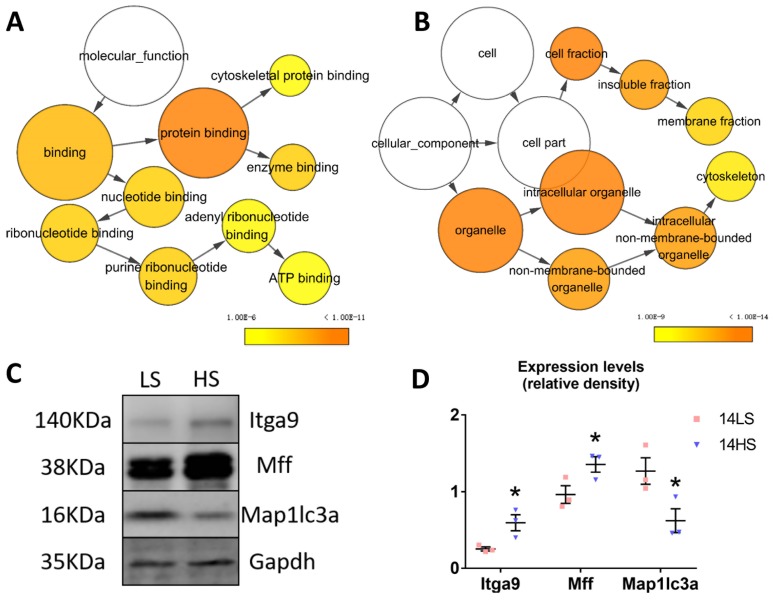
GO analyses and validation of dysregulated proteins identified by proteomics. (**A**,**B**) GO analysis of a gene network constructed from 80 differential expression proteins (21 significantly downregulated and 59 significantly upregulated proteins). The color gradient of the cluster distribution network shows the *p*-value of each cluster with darker (orange) color for a lower *p*-value. (**A**) Molecular functions and (**B**) cellular components. (**C**) Western blot results showing expression levels of Itga9, Mff, and Map1lc3a in the two groups, where all data were normalized to Gapdh expression. (**D**) Statistical analysis of the intensity of the bands in Figure 6C using ImageJ software (*n* = 3 for each group). * *p* < 0.05.

Shotgun proteomic analysis obtained 21 significantly downregulated and 59 significantly upregulated proteins from a total of 3701 proteins in the HS group as compared with the LS group. Figure 5A shows a heatmap of the proteome, where hierarchical clustering of samples (columns) and proteins (rows) is based on Pearson’s correlation coefficient to measure the distance and the mean to cluster the samples. There was significant difference of proteomes between HS-fed and LS-fed rat hearts (Figure S1). Figure 5B shows Gene Ontology (GO) analysis on the 80 differentially expressed proteins using DAVID Bioinformatics Resources 6.8, which identified mitochondrial fission and organelle fission as the top upregulated biological processes, and microtubule binding and GTPase activity as the top upregulated molecular functions, while prostaglandin-endoperoxide synthase (COX) and regulation of blood pressure were the top downregulated biological processes. Figure 6A,B shows GO analysis of a gene network constructed from the 80 differentially expressed proteins using the BinGO plugin (Figure S2). We found that HFpEF was highly correlated with molecular functions (e.g., cytoskeleton protein binding, enzyme binding, nucleotide binding, ribonucleotide binding, and ATP binding) as well as multiple cellular components (e.g., cell fraction, insoluble fraction, membrane fraction, intracellular organelle, and cytoskeleton). Gene Set Enrichment Analysis (GSEA) detects more biology-driven gene sets of canonical pathways from the molecular signature database (MSigDB). As shown in Figure 5C and Supplementary Table S3, gene sets that were significantly downregulated in the HS group at *p*-value < 0.01 and false discovery rate (FDR) < 0.05 included complement and coagulation cascades, cardiac muscle contraction, spliceosome, and so on, while upregulated gene sets were fructose and mannose metabolism and proteasome. To validate the proteomic data, three differentially expressed proteins, Integrin α9 (Itga9), Mff, and Map11c3a, which play important roles in regulating cytoskeleton and mitochondrial function, were chosen, and their expression was detected by Western blot. Results showed significant up-regulation of Mff and Integrin α9 and down-regulation of Map1lc3a in rat hearts of HFpEF in comparison with the controls, as shown in Figure 6C,D.

### 2.6. Microarray Study and Quantitative Polymerase Chain Reaction

**Figure 7:**
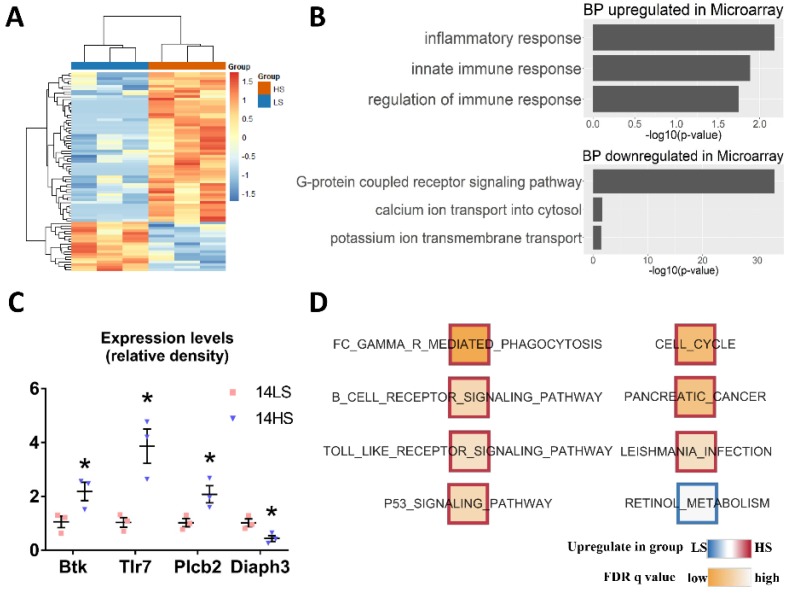
GO, GSEA, and real-time PCR analyses on the dysregulated genes identified by transcriptome. (**A**) Heatmap of the transcriptome, where hierarchical clustering of samples (columns) and genes (rows) is based on Pearson’s correlation coefficient to measure the distance and the mean to cluster the samples. (**B**) GO Analysis of 465 significant downregulated and 142 upregulated genes. (**C**) Real-time PCR results showing expression levels of Btk, Tlr7, Plcb2, and Diaph3 in the two groups, where all data were normalized to Gapdh expression. Fold change for expression level comparison was calculated with 2^(-ddCT). Primers are listed in Supplementary Table S1. * *p* < 0.05. (**D**) GSEA of all detected genes, where nodes represent significantly enriched (*p* < 0.01, *q* < 0.05) gene sets of canonical pathways from the MSigDB. The colors of node borders refer to upregulated (red) and downregulated (blue) in HS group, while the node color represents the FDR q-values.

The microarray analysis obtained 465 significantly downregulated and 142 significantly upregulated genes from a total of genes in the HS group as compared with the LS group. Figure 7A and Figure S1 show heatmap analysis of the transcriptome demonstrating significant differences between HS-fed and LS-fed rat hearts. Figure 7B shows GO analysis of the 607 differential expression microarrays using the DAVID Bioinformatics Resources 6.8, which identified inflammatory response, innate immune response, and regulation of immune response as the top upregulated biological processes, and G protein-coupled receptors as the top downregulated biological process. Among the dysregulated genes, Btk, Tlr7, Plcb2, and Diaph3 play an important role in B-cell development, pathogen recognition and activation of innate immunity, intracellular transduction of many extracellular signals, and regulation of cell movement and adhesion. Their expression levels were verified by real-time PCR. Btk, Tlr7, and Plcb2 were significantly up-regulated, and Diaph3 was down-regulated in rat hearts of HFpEF compared with the controls (Figure 7C). Moreover, anti-apoptotic factor Bcl2l10 was significantly downregulated, while pro-apoptotic factors Bcl2l14 and Bax were upregulated in rat hearts of HFpEF. Gene sets that were significantly downregulated in the HS group at *p*-value < 0.01 and FDR < 0.05 included retinoid metabolism, while upregulated gene sets were Fc-gamma receptors, cell cycle, and so on (Figure 7D and Supplementary Table S4).

## 3. Discussion

Since HFpEF is multifactorial in nature involving the interactions of environmental, physiological, and genetic risk factors, various experiments were carried out to diagnose HFpEF in HS-fed DSS rat hearts, while histological and multi-omics analyses were performed to address cellular and molecular mechanisms. Here, we report several findings: (1) HS-fed rats with hypertension and myocardial hypertrophy developed diastolic dysfunction, impaired systolic contractile function, and prolonged myocyte repolarization despite preservation of EF. (2) A significant increase in size and apoptosis of myocytes occurred in HS-fed rat hearts as compared with the LS-fed. (3) Bioinformatics analyses showed that genes involving in autophagy and cardiac contraction were significantly downregulated, while genes regulating mitochondrial fission and inflammatory processes were significantly upregulated in HS-fed rats. These findings are discussed as follows.

### 3.1. Pathophysiological Changes in Organ and Tissue Levels

HS-fed and LS-fed DSS rats were characterized with equivalent values of EF and QRS width at the ages of 7 and 14 weeks, which agrees with a previous study [11]. In contrast, HS feeding increased E/E’ ratio, QT interval and QTc interval, isovolumetric relaxation time (Tau), blood pressure (ASP, ADP, MAP), and LVEDP, while it decreased E/A ratio, peak radial, and longitudinal strains in the LV. This revealed the progression of diastolic dysfunction, impaired systolic contractile function, and prolonged myocyte repolarization in DSS rats after 7 weeks of a HS diet. HS-fed DSS rats also showed HF symptoms including weakness and decreased activity. A significant increase of lung weight to BW implies pulmonary edema in HS-fed DSS rats. Based on the diagnostic criteria [2,24,25], HFpEF occurred in HS-fed DSS rats. On the other hand, HS-feeding resulted in severe LV hypertrophy because of a significant increase in LV weight/BW, BNP and TIMP-1 mRNA level, and myocyte size. This contributes to myocardial and myocyte stiffening and, hence, impairs both diastolic and systolic contractile functions, consistent with previous findings [26,27,28].

### 3.2. Promising Regulatory Gene Targets for Myocardial and Myocyte Stiffening

The Heatmap of the proteome and transcriptome showed significant differences between HS-fed and LS-fed rat hearts. Based on the bioinformatics analysis, the downregulated gene set of cardiac muscle contraction and biological process of Ca^2+^ ion transport into cytosol in the HS group indicated the effects of Ca^2+^-mediated signaling on impairment of active diastolic relaxation in HFpEF [29,30]. Both transcriptome and real-time PCR data showed that Plcb2 was significantly upregulated and Diaph3 was downregulated (Figure 7). Phospholipase C (PLC) produces inositol 1,4,5-triphosphate (IP3) and diacylglycerol from phosphatidylinositol 1,4,5-biphosphate, leading to many downstream events including intracellular calcium and activation of protein kinase [31]. PLC-βs have important roles in chemoattractant-induced integrin activation, affecting cell–substrate adhesion and migration [32,33]. As a member of PLC-βs, Plcb2 was upregulated to mediate Ca^2+^ release that depolarizes the plasma membrane to generate action potentials and subsequent non-vesicular release of ATP [34]. Furthermore, as a member of the formin family of cytoskeletal regulators, diaphanous-related formin-3 (Diaph3) regulates cytoskeleton formation, cell adhesion, migration, and differentiation [35]. Downregulation of Diaph3 can restrict actin remodeling [36], which could regulate movement of myocytes. Therapies targeting these Ca^2+^ and cytoskeleton-mediated genetic risk factors may merit investigations for elevated myocyte stiffness in HFpEF.

Previous studies have demonstrated marked interstitial fibrosis [37,38,39] and decreased capillary density in rat hearts of HFpEF [39]. An increase in collagen deposition could stiffen the heart, resulting in impaired diastolic function [40], whereas low capillary density could deprive the oxygenation of hypertrophic myocytes [41]. Similarly, HFpEF patients displayed exercise-mediated reductions [42], coupled with varying degrees of myocardial disease progression including myocyte hypertrophy, interstitial fibrosis, and capillary rarefaction [43].

A high prevalence of comorbidities such as agedness, hypertension, and obesity in HFpEF patients [44] could stimulate systemic inflammation that causes myocardial dysfunction and remodeling in HFpEF patients [45,46]. HFpEF rat hearts showed molecular signatures of immune cell and inflammatory dysregulation, which is a distinct different characteristic compared to HFrEF [47,48]. High inflammatory marker levels had a strong association with HFpEF. This association is explicable because inflammation has been linked with diastolic dysfunction in hypertensive patients [49], and inhibition of inflammatory pathways prevents diastolic dysfunction in experimental diabetic cardiomyopathy [50]. The downregulated gene sets of complement and coagulation cascades, the upregulated gene sets of Fc-gamma receptors, B-cell receptors, and Toll-like receptors, the upregulated biological processes of inflammatory response, and innate immune response in the HS group denoted an important role of the inflammatory response in HFpEF. As key signaling genes controlling the activation of various immune cells, the upregulation of Btk and Tlr7 may be involved in activating profibrotic and hypertrophic cascades and further deteriorating myocardial stiffening in HFpEF [1].

In summary, Ca^2+^-mediated signaling, cytoskeleton formation, interstitial fibrosis, capillary rarefaction, and inflammation might be critical therapeutic targets for HFpEF, for which no effective therapy currently exists. Further investigations were required to prove agents with calcium regulation, cytoskeleton organization, or anti-inflammatory properties can be able to modulate HFpEF.

### 3.3. Promising Regulatory Gene Targets for Myocyte Apoptosis

TUNEL staining showed an increase of apoptosis rate of myocytes in HS-fed rats as compared with LS-fed rats in Figure 4, which is supported by the microarray results of pro- and anti-apoptotic factors (e.g., downregulated Bcl2l10 and upregulated Bcl2l14 and Bax). Both proteomic and Western blot data showed that Mff and Itga9 were significantly upregulated, but Map1lc3a was downregulated in HS-fed rat hearts (Figure 6). Mitochondrial fission factor (Mff) is a protein to control mitochondrial fission, integrin α9 is a protein to mediate cell–cell and cell–matrix adhesion, and microtubule-associated proteins 1A/1B light chain 3A (Map1lc3a) is a protein to regulate autophagy functions.

Mitochondrial dynamics comprises fusion and fission, which produces an interconnected mitochondrial network and results in smaller, more discrete organelles, respectively. Two mitofusin isoforms, Mfn1 and Mfn2, and optic atrophy protein 1 (OPA1) are core components of the mitochondrial fusion machinery, while mitochondrial fission is regulated by a dynamin-related protein 1 (Drp1). Mff, fission protein 1 (Fis1), and Mitochondrial dynamics proteins of 49/51 kDa (MiD49/51) have been proposed to promote mitochondrial fission by recruiting Drp1 to mitochondria. There is a significant upregulation of Mff in rat hearts of HFpEF as compared with the controls, but relatively unchanged expression of Mfn1, Mfn2, OPA1, Fis1, and MiD49/51 based on proteomics results. Dysfunctional mitochondria caused by excessive Mff can be removed by the process called mitophagy [51]. However, Map1lc3a, a gene tethering autophagosome to mitochondrion, is significantly downregulated to decrease clearance of the damaged mitochondria. This probably leads to accumulation of aberrant mitochondria to increase ROS and impair calcium homeostasis, and subsequent contractile dysfunction and myocardial apoptosis [52,53,54,55]. Moreover, a previous study reported that NADPH oxidase activity, xanthine oxidoreductase (XOR) activity, and superoxide levels in rat hearts of HFpEF were significantly higher than the controls [38]. These findings denote Mff and Map1lc3a abnormalities as potential genetic risk factors for increased apoptosis rate of myocytes in HFpEF. In addition, GO analysis of 80 differential expression proteins also showed microtubule binding and GTPase activity as the top upregulated molecular functions, which may result in more dysfunctional mitochondria.

In animal models and human studies of HF, dysfunctional mitochondria exhibit decreased ATP production and marked excess reactive oxidative stress (ROS) production [56]. Cardiac mitochondria dysfunction in HFpEF patients also causes overexpression of proinflammatory cytokines [57]. The accumulation of abnormal mitochondria derives ROS and acts as principal arbitrators of pro-inflammatory status. They act through modulating innate immunity via redox-sensitive inflammatory pathways or direct activation of inflammasome such as NLRP3. Additionally, both of these pathways may work together to overstimulate the inflammatory response [58].

In this study, we speculate the accumulation of aberrant mitochondria and inflammatory response may cause excessive ROS production and impair calcium homeostasis, and ultimately increase interstitial fibrosis and cause cardiac remodeling in HS-fed rats. Hence, investigating compounds that target these pathways is promising for future treatment of HFpEF.

### 3.4. Critique of the Study

Although DSS rats are the proven and most published animal model of HFpEF [52,53,54,55], the model only represents a minority of patients suffering from salt-sensitive hypertension. This requires further investigations in other animal models. The upstream signaling pathways, however, remain unknown, which may be an important external stress for the occurrence and development of HFpEF. Cardiac tissues were harvested for the multi-omics analysis to gain an overview of the changes in HFpEF, which brought in the influence of interstitial cells. Isolation of myocytes is still required to accurately study HFpEF molecular networks.

## 4. Materials and Methods

### 4.1. Study Design

The rat model of HFpEF was established as shown schematically in Figure 1A, similar to previous studies [10,11]. Briefly, 7-week-old DSS male rats (purchased from Beijing Vital River Laboratory Animal Technology Co., Ltd., Beijing, China) were randomly classified into a HS diet group (SPF Rodent Growth and Breeding Feed + 8% NaCl with irradiation, Beijing Keao Xieli Feed Co., Ltd., Beijing, China) to induce HFpEF (*n* = 17), or into a low-salt (LS) diet group (0.3% NaCl with irradiation) as controls (*n* = 10). Rats were housed individually in air-conditioned facilities at room temperature, under 12:12 h light/dark artificial cycle conditions, and supplied with different diets for 7 weeks. Most HS-fed rats showed HF signs including weakness, decreased activity, and labored breathing at the age of 14 weeks. Echocardiographic and electrocardiographic results demonstrated whether symptomatic HS rats had HFpEF. Furthermore, since eight LS rats underwent hemodynamic measurements, given two died during the operation, eight HS rats showing diastolic dysfunctions and successfully having hemodynamic measurements were selected for histological evaluation and multi-omics analysis. Body and organ weights were obtained from these animals. In each group, five animals were used for histological evaluation, while three animals were dissected to harvest cardiac tissues for microarray and proteomics study. All experiments were performed in accordance with Chinese National and Peking University ethical guidelines regarding the use of animals in research, consistent with the NIH guidelines (Guide for the care and use of laboratory animals) on the protection of animals used for scientific purposes. The experimental protocols were approved by the Animal Care and Use Committee of Peking University, China, agreement number COE-HUOYL-1 (14 Jul 2014).

### 4.2. Echocardiographic Measurements

Rats were anesthetized with inhalational administration of 5% isoflurane and maintained with 2% isoflurane for echocardiography. Echocardiographic measurements of rat hearts were carried out in the two groups before and after the experiment, similar to a previous study [59]. The images were obtained at 21 MHz using a MS-250 transducer operated by a Vevo2100 Color Doppler Ultrasound Scanner (FUJIFILM VisualSonics Inc., Toronto, ON, Canada). Based on M-mode tracings, morphometric parameters, e.g., LVID;d and LVID;s, were measured according to the American Society of Echocardiography leading edge rule [60]. These parameters were averaged based on five measurements. EF (%) was calculated from the measured parameters as $\frac{\left({\mathrm{LVID};d}^{3}{-\mathrm{LVID};s}^{3}\right)}{{\mathrm{LVID};d}^{3}}\times 100\%$ in Vevo2100 LAB image analysis workstation [61]. Details are in the supplement section.

### 4.3. Surface Electrocardiogram

Surfaced electrocardiograms (ECG) were recorded in animals of the two groups. QRS complex and RR interval were measured to assess systolic functions, while corrected QT (QTc) interval (i.e., QT interval (ms) divided by the square root of RR interval (s)) was used to evaluate diastolic functions. All ECG data were averaged over nine cardiac cycles by a waveform-analysis program (SP2006, Softron, Beijing, China).

### 4.4. Speckle Tracking Echocardiography (STE) Measurements

In B-mode tracings, 2D grayscale images were obtained from the standard parasternal longitudinal view [62]. Frame rate was 133 Hz, gain was 20~25 dB, depth was ~20 mm, width was ~23 mm, and three cardiac cycles were recorded. Myocardial deformations were demonstrated using the Vevo2100 LAB image analysis workstation with advanced STE, which tracks natural acoustic markers (called speckles) across the cardiac cycle. Longitudinal and radial stains ($=\frac{∆L}{L_{0}}$, where $L_{0}$ and ∆L refer to the baseline length at the R wave and the absolute change in length, respectively) were determined by the software across the entire left ventricle over selected period of cardiac cycles.

### 4.5. Doppler-Mode Tracings

Diastolic function was assessed by E/E’ and E/A ratios in the apical four-chamber view, as shown in Figure 1C. E waves (early filling) and A waves (atrial filling) were measured by pulse wave Doppler mode between the mitral valves. E’ waves and A’ waves were measured at the septal corner of the mitral annulus by tissue Doppler mode.

### 4.6. Hemodynamic Measurements

Hemodynamic analysis was carried out on rats anaesthetized with 2% isoflurane. A 1.4F micromanometer-tipped catheter (Millar Instruments, Houston, TX, USA) was inserted through the right carotid artery into the LV to record pressure waves in 30 cardiac cycles, which was repeated three times. The zero-pressure baseline of the catheter was calibrated in 37 °C saline. The catheter was monitored with a BIOPAC MP150. LV end-diastolic pressure (EDP), rate of maximum and minimum left ventricular pressure development (dP/dt max, dP/dt min), and time constant of LV relaxation (Tau); artery systolic, Diastolic, and mean pressure (ASP, ADP, AMP) were determined from the measured pressure waves [61].

### 4.7. Histological Evaluation

As shown in Figure 1A, animals were separately terminated for the histological evaluation at the age of 14 weeks (i.e., HS-feeding or LS-feeding for 7 weeks). After hearts were harvested, plugs of myocardial tissues were removed from different positions of the LV. These plugs were fixed in 4% paraformaldehyde (PFA)/PBS solution overnight at room temperature and then processed for paraffin sectioning. TUNEL staining was demonstrated according to the manufacturer’s recommendations (In Situ Cell Death Detection Kit, Fluorescein, catalog number 11684795910, Roche, Basel, Switzerland) [63]. Sections were also detected via Alexa Fluor 594 conjugate of WGA (50 μg/mL, Invitrogen, Carlsbad, CA, USA), similar to a previous study [64]. Moreover, nuclear morphology was assessed by Hoechst 33258 dye (Molecular Probes) as reported [65].

### 4.8. Shotgun Proteomics Analysis

Similar to a previous study [66], proteins were extracted from the harvested cardiac tissues using RIPA Lysis Buffer and PMSF (Applygene, Beijing, China) in glass homogenizers. Once homogenized, cardiac tissues were centrifuged at 12,000 *g* at 4 °C for 20 min, and supernatants were collected for the shotgun proteomics analysis. The protein concentration of the supernatant was evaluated using the BCA protein assay kit (Applygene). A total of 200 μg protein was reduced with 0.05 M TCEP, alkylated with iodoacetamide, and digested by 4 μg of trypsin. A nano-flow HPLC instrument (Easy-nLC II) coupled with an LTQ-Orbitrap Velos Pro (linear quadrupole ion trap-orbitrap mass analyzer) mass spectrometer equipped with a Nanospray Flex Ion Source (Thermo Fisher Scientific, Waltham, MA, USA) was used for the LC-MS/MS analysis. Each peptide from the LC-MS/MS spectra was searched against UniProt rat protein database using MaxQuant software. False discovery rate was calculated by decoy database searching, and differentially expressed proteins were identified by fitting the data to a linear model with an empirical Bayes moderated *t*-statistics test.

### 4.9. Microarray Analysis

Myocardial tissues were washed by injecting PBS into the left ventricle in vivo. Agilent Whole Rat Genome Oligo microarray experiments were performed by KangChen biotech company (Shanghai, China). The subsequent steps were conducted according to the Agilent Whole Genome Oligo microarray (one-color) protocol. The accession number for the microarray data reported in the study is GEO GSE126062.

### 4.10. Bioinformatics

For shotgun proteomics and microarray analysis, differentially expressed genes and proteins were filtered at *p* < 0.05 and the absolute value of log2 (fold change) > 1. Gene Ontology (GO) analysis for differentially expressed proteins and genes was performed using the DAVID Bioinformatics Resources 6.8 (https://david.ncifcrf.gov/). The protein-to-protein network was expended using BisoGenet plugin in Cytoscape environment. The BiNGO plugin was used to retrieve the Gene Ontology Consortium. Moreover, Gene Set Enrichment Analysis (GSEA) was made for the KEGG pathway enrichment analysis based on the whole gene set with the rank list of all the available expression values. Proteins with more than two unique peptides from LFQ intensities were defined as qualified proteins for GSEA. C2.cp.kegg from the Molecular Signatures Database (MSigDB, v6.2) was selected as gene sets. Significantly enriched pathways were selected at a level of 0.01 normal *p*-value and 0.05 FDR q-value. Subsequently, these pathways were subjected to the Enrichment Map plugin in the Cytoscape environment (https://cytoscape.org/).

### 4.11. Western Blot

Equal protein amounts (100 μg) were boiled in loading buffer at 100 °C for 5 min and run in 10% SDS-polyacrylamide. After electrophoresis, gels were electrotransferred onto PVDF membranes, blocked 1h at room temperature with 5% nonfat dried milk in TBST (Tris-buffered saline with 0.1% Tween-20), and incubated at 4 °C overnight with primary antibodies following standard commercial instructions. The primary antibodies were used as follows: mouse anti-Gapdh (1:1000 diluted, Proteintech), rabbit anti-ITGA9 (1:5000 diluted, ab140599, Abcam, Cambridge, UK), rabbit anti-MAP1lC3A (1:5000 diluted, ab52638, Abcam), and rabbit anti-MFF (1:500 diluted, 17090-1-AP, Proteintech, Rosemont, IL, USA). Secondary antibodies used were goat anti-rabbit (1:5000 diluted, ZB-2301, Zsbio and 611145002, Rockland Immunochemicals, Gilbertsville, PA, USA) anti-rabbit and goat anti-mouse (1:5000 diluted, 610144002, Rockland Immunochemicals). Images were scanned on LiCOR Odyssey.

### 4.12. Quantitative RT-PCR

Total RNA from myocardial tissues was extracted using RNAtrip kit (Applygene). The quality and quantity were measured by a nanophotometer (IMPLEN, Beijing, China). RNA was used for reverse transcription using the RevertAid First Strand cDNA Synthesis Kit (Thermo Fisher Scientific). Diluted cDNA (10×) was made for quantitative RT-PCR reactions using the QuantStudioTM3 (Thermo Fisher Scientific) and a HieffTM qPCR SYBR Green Master Mix (Yeasen, Shanghai, China). All data were normalized to the Gapdh expression. Fold change for expression level comparison was calculated with 2^(-ddCT). Primer sets for quantitative real-time PCR analyses were designed using Primer Premier 5, as shown in Supplementary Table S1.

### 4.13. Statistical Analysis

All data analysis and plots were generated using R Studio and GraphPad Prism 6.0. For the rest of the analysis, data were expressed as mean ± standard error (SE), and *p*-value was calculated using the two-tailed Student’s *t*-test for pairwise comparisons. Statistical tests were carried out using GraphPad Prism 6.0, San Diego, CA, USA (*, *p* < 0.05; **, *p* < 0.01; ***, *p* < 0.001).

## 5. Conclusions

Most DSS rats when placed on a HS diet for 7 weeks showed HF symptoms and were diagnosed with HFpEF by echocardiographic, electrocardiographic, and hemodynamic measurements. In comparison with LS-fed DSS rats, rats with HFpEF demonstrated a significant increase in size and apoptosis rate of myocytes. Upregulated Mff and downregulated Map1lc3a were found as potential risk factors for increased apoptosis rate of myocytes in HFpEF. The changes of multiple molecular functions such as cytoskeleton protein binding, ATP binding, and microtubule binding also contributed to diastolic activity loss and myocyte stiffening. This study sheds light on therapies targeting these risk genetics.

## Figures and Tables

**Table 1:** LV diastolic and systolic diameters and ratios of heart, LV, lung, and kidney weights to BW in HS and LS groups at the age of 14 weeks.

Group	No.	LVEDD (mm)	LVESD (mm)	LV/BW (g/kg)	Heart/BW (g/kg)	Lung/BW (g/kg)	Kidney/BW (g/kg)
14LS	8	7.10 ± 0.10	3.80 ± 0.25	2.37 ± 0.10	3.60 ± 0.11	3.96 ± 0.05	3.73 ± 0.16
14HS	8	7.17 ± 0.23	4.11 ± 0.43	3.42 ± 0.26 **	4.06 ± 0.15 *	4.18 ± 0.06 *	4.31 ± 0.08 **

LVEDD indicates left ventricular end diastolic diameter; LVESD indicates left ventricular end systolic diameter; and * *p* < 0.05, ** *p* < 0.01.

**Table 2:** Hemodynamic parameters in HS and LS groups at the age of 14 weeks.

Group	No.	LVEDP (mmHg)	dP/dt max (mmHg/s)	dP/dt min (mmHg/s)	ASP (mmHg)	ADP (mmHg)	MAP (mmHg)
14LS	8	3.72 ± 0.38	9895 ± 833	−6890 ± 528	160.5 ± 7.9	106.5 ± 7.7	124.5 ± 6.9
14HS	8	11.14 ± 2.03 **	7010 ± 941 *	−5051 ± 489 *	184.6 ± 6.8 *	128.2 ± 4.4 *	147.0 ± 4.9 *

* *p* < 0.05, ** *p* < 0.01.

## Supplementary Materials

Supplementary Materials can be found at https://www.mdpi.com/1422-0067/21/9/3362/s1.

## Abbreviations

HFpEF	Heart failure with preserved ejection fraction
HFrEF	Heart failure with reduced ejection fraction
EF	Ejection fraction
LV	Left ventricular
DSS	Dahl salt-sensitive
HS	High-salt
LS	Low-salt
GSEA	Gene Set Enrichment Analysis
GO	Gene Ontology
