# Supplementary material for: Morphometric, Hemodynamic, and Multi-Omics Analyses in Heart Failure Rats with Preserved Ejection Fraction
Source: Int J Mol Sci. 2020 May 9;21(9):3362. doi: 10.3390/ijms21093362 (PMC7247709; doi:10.3390/ijms21093362)
Supplement: Supplementary file 1 [file ijms-21-03362-s001.pdf]

Supplementary Table S1. Primer sets for quantitative real-time PCR.

Gene	Forward	Reverse
Bnp	TTCTGCTCCTGCTTTTCC	GAGCCATTTCCTCTGACTTT
Timp1	AGAGCAGATACCACGATG	GTCCGAGTTGCAGAAAGC
Btk	AGCACCAATCTCCACAA	AATACTCCTCGCCCTTTCG
Tlr7	GTTTTACGTCTACACAGTAACTCTCTTCA	TTCCTGGAGGTTGCTCATGTTTT
Plcb2	GTGACCGAGCAAATCGCCAAGA	CTTTGGTGTCAGTTTCCGAGGT
Diaph3	TAAATGCCCTTGTTACATCTCC	TTCAATCCACAACGCATAAA
Gapdh	ATGACCACAGTCCATGCCAT	TTCAGCTCTGGGATGACCTT
Bnp	TTCTGCTCCTGCTTTTCC	GAGCCATTTCCTCTGACTTT

Supplementary Table S2. BNP and TIMP-1 mRNA levels.

Group	N	LS	HS
BNP	4	1.04± 0.17	2.34± 0.33*
TIMP1	4	1.16 ± 0.39	3.73 ± 0.82*

*P<0.05

Supplementary Table S3. Details of the gene sets enriched by GSEA in proteomics.

Group	NAME	SIZE	NES	NOM p-val	FDR q-val
LS	PARKINSONS DISEASE	68	-2.08	0	0.021
LS	COMPLEMENT AND COAGULATION CASCADES	37	-2.06	0	0.013
LS	ALZHEIMERS DISEASE	84	-1.95	0	0.033
LS	CARDIAC MUSCLE CONTRACTION	39	-1.93	0	0.032
LS	HUNTINGTONS DISEASE	87	-1.85	0	0.045
LS	SPLICEOSOME	58	-1.84	0.003	0.042
HS	PROTEASOME	38	1.80	0.002	0.044
HS	FRUCTOSE AND MANNOSE METABOLISM	23	1.77	0.009	0.038

Supplementary Table S4. Details of the gene sets enriched by GSEA in microarray.

Group	NAME	SIZE	NES	NOM p-val	FDR q-val
LS	RETINOL METABOLISM	31	-1.90	0	0.019
HS	FC GAMMA R MEDIATED PHAGOCYTOSIS	91	2.11	0	0.002
HS	CELL CYCLE	117	1.97	0	0.006
HS	PANCREATIC CANCER	67	1.94	0	0.007

HS	P53 SIGNALING PATHWAY	60	1.90	0	0.012
HS	LEISHMANIA INFECTION	54	1.88	0	0.013
HS	B CELL RECEPTOR SIGNALING PATHWAY	71	1.87	0	0.012
HS	TOLL LIKE RECEPTOR SIGNALING PATHWAY	85	1.84	0	0.014

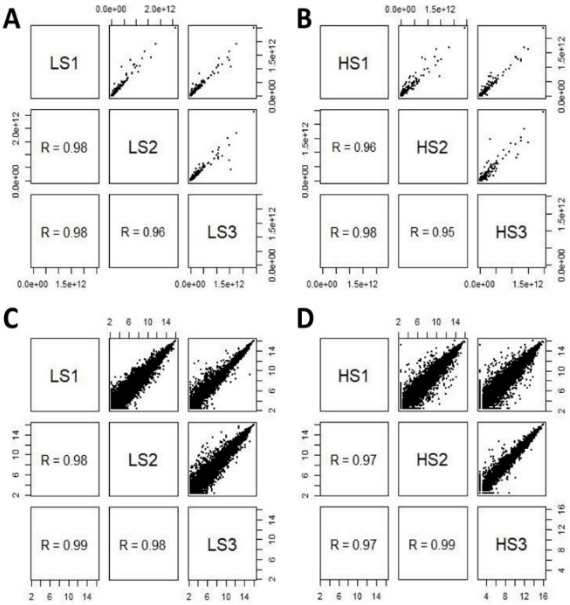

Figure S1. Reproducibility correlation matrices of proteomics (A and B) and microarray data (C and D), linear regression, n=3/group.

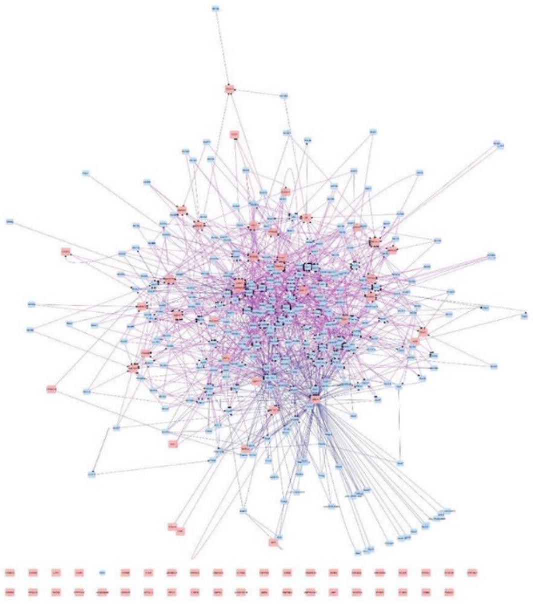

Figure S2. Protein-to-protein interaction network expanded from all differentially expressed proteins using BinGO plugin in the Cytoscape environment.
